# A Graph-Theoretic Approach for Identifying Non-Redundant and Relevant Gene Markers from Microarray Data Using Multiobjective Binary PSO

**DOI:** 10.1371/journal.pone.0090949

**Published:** 2014-03-13

**Authors:** Monalisa Mandal, Anirban Mukhopadhyay

**Affiliations:** Department of Computer Science and Engineering, University of Kalyani, Kalyani, West Bengal, India; University of Bonn, Bonn-Aachen International Center for IT, Germany

## Abstract

The purpose of feature selection is to identify the relevant and non-redundant features from a dataset. In this article, the feature selection problem is organized as a graph-theoretic problem where a feature-dissimilarity graph is shaped from the data matrix. The nodes represent features and the edges represent their dissimilarity. Both nodes and edges are given weight according to the feature’s relevance and dissimilarity among the features, respectively. The problem of finding relevant and non-redundant features is then mapped into densest subgraph finding problem. We have proposed a multiobjective particle swarm optimization (PSO)-based algorithm that optimizes average node-weight and average edge-weight of the candidate subgraph simultaneously. The proposed algorithm is applied for identifying relevant and non-redundant disease-related genes from microarray gene expression data. The performance of the proposed method is compared with that of several other existing feature selection techniques on different real-life microarray gene expression datasets.

## Introduction

Data dimensionality reduction can be done in two ways: 1) feature extraction creates new feature by combining features and, 2) feature selection choose subset of features by eliminating features with less or no predictive information. The center of attention of this proposed study is only on the feature selection. Feature selections have immense impact in improving the quality of classification and clustering technique in machine learning and pattern classification. The feature selection can be applied to both supervised and unsupervised learning. In a supervised scenario [1], [2], the correct class of all training samples are additionally known and the feature evaluation criteria to generate selected feature set are based on the known class label of the features. In contrast, in unsupervised cases the assessment criteria are completely independent of the true class labels of the features. Performance in unsupervised classification is typically considered as the capability of a clustering algorithm to expose groupings (clusters) in a given data set. Subsequently, the clustering solution is evaluated using some cluster validation techniques like entropy (E), class separability (S), fuzzy feature evaluation index (FFEI), etc [3]. Again feature selection may be filter-based or wrapper-based approach. When the utility of a feature is measured in terms of some proxy measure, then it is called filter-based feature selection. The proxy measure uses the class label in supervised filter-based approach. In unsupervised filter, the proxy measure considers the degree to which the distribution of the feature values exhibits the class structure in the feature space. Utility measures for wrapper methods [2] completely rely on a classifier or clustering result. As filter methods are independent of the classifier applied subsequently, they have excellent generalization properties, but may be less effective at decreasing the dimensionality of the feature space and boosting classification accuracy. Generally, they are computationally cheaper than the wrapper approaches. But wrapper based methods are more prone to have data over-fitting. The variety of feature selection technique has been addressed in quite a few ways such as clustering based [4], [5], content based [6], for ensemble classifier [7], graph based [8], [9] and feature similarity based [3].

In this context, two opposite strategies have been proposed in the literature: those that aim at the exclusion of redundant features [3] and those that focus on the elimination of irrelevant features [10]. Besides these methods there exist some Particle Swarm Optimization (PSO) based feature selection techniques in the literature. In [11], a multiswarm binary PSO has been introduced. A scheduling algorithm has been executed for selecting fittest subswarm where classification accuracy and fscore are combined as objective function. Then in [12], author used PSO and Least Square Support Vector Machine for feature selection and in [13] an improved PSO with signtest has been described for identifying relevant features. Again article [14] used bPSo but all these methods have been modeled as single objective fashion where classification accuracy has been considered as objective function. However, also there exist multiobjective PSO-based approaches like [15], [16] and [17] where MOPSO has been well studied but they did not consider the redundancy among features which should be minimized for reducing computation cost and improving the performance. Therefore, the objective of feature selection should be to select the most significant or relevant as well as non-redundant features.

In this article we have proposed a novel graph-theoretic model for selecting most relevant and non-redundant features from the input dataset. In the proposed method, first a complete graph is shaped where the nodes symbolize the features and edge weights are defined by the dissimilarity among the features. Then we extract the densest subgraph from the feature-dissimilarity graph. The attributes contained by the extracted subgraph comprise the final selected relevant and non-redundant features. For identifying the densest subgraph, we have projected a multiobjective binary particle swarm optimization (MO-bPSO) based algorithm. The particles are fashioned as binary strings for encoding the feature subset. Two objective functions, average node-weight and average edge-weight are optimized simultaneously. Unlike single objective optimization which yields a single best solution, multiobjective optimization (MOO) [18], [19] algorithms turn out a set of solutions which contains a number of non-dominated solutions, none of which can be further improved on any one objective without degrading it in another. Here the multiobjective optimization problem is tackled by applying bPSO [20] in which fitness comparison takes Pareto dominance [21] into account during the movement of the particles in the search space. The non-dominated solutions are stored in an archive to approximate the Pareto front [22].

In this proposed article, feature selection technique is applied to identify relevant and non-redundant gene markers from microarray gene expression data [23]. Microarray is a rapidly growing technology that provides the opportunity to assay the expression levels of genes in a single experiment. A microarray gene expression data set contains the expression levels of thousands of genes over a number of tissue samples. Hence this is a sample versus gene matrix which also contains the class label for each sample. Although recently it has gained popularity in the process of finding disease-related gene or marker, its high dimensionality and noise pose a challenging problem. Moreover some genes may not be very relevant to the corresponding class labels; hence they are not helpful for phenotype classification. In binary classification [24], the task of classification is done to the samples of the microarray dataset consisting of normal (benign) and cancer (malignant) tissue. Otherwise when samples represent three or more subtypes of cancer then classification [25] is called multiclass cancer classification.

It is common in practice that in order to find the most relevant genes, most of the existing feature selection techniques [26], [27] produce a redundant set of genes. This fact has encouraged us to apply our proposed graph-based multiobjective binary particle swarm optimization technique which selects not only the relevant genes but a non-redundant set of genes also. The performance of the proposed technique is established on different real-life microarray gene expression data sets and compared with that of various existing gene selection techniques.

## Materials and Methods

### Other Relative Methods

There are many more feature selection techniques in the existing literature establish their own superiority. In this article, we have taken some of them namely, T-test, Ranksum test, SFS, SBE, CFS, mRMR(MIQ), Graph-based feature selection () and Cluster-based feature selection(). Moreover as our method is multiobjective one, so the singleobjective versions are also taken into account. By nature, the Sequential Forward Search (SFS) [28] selects features sequentially depending on the adopted criteria. On the contrary, Sequential Backward elimination (SBE) [29] discards features on the basis of the adopted criteria. Additionally, a methods like Correlation-based Feature Selection (CFS) [30] has been used for performance analysis. Here, the ratio of snr value to mean correlation value is considered as the criteria to calculate the features importance. The number of resultant features of our proposed approach is the input of the other comparative algorithms like T-test, Ranksum test, SFS, SBE, CFS and mRMR(miq). In case of T-test [31], and Ranksum test [32], [26], at first the p-values of the features are sorted and required numbers of features are taken for validation. In mRMR feature selection technique [33], [34], the relevance of gene is calculated by mutual information [35] between a feature and its corresponding class labels and redundancy is computed as the mutual information among the features. The basic concept of mRMR is to select the genes such that they are relevant and mutually maximally dissimilar to each other at the same time. Let *s* denotes the subset of genes that we are seeking. The average minimum redundancy is given as Equation 1:
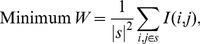
(1)where 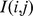 presents the mutual information between *i*-th gene and *j*-th gene and 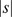 is the number of genes in *S*. The discriminant power of a gene by the mutual information 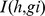 is calculated as per Equation 2. That means the mutual information between targeted classes 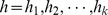 and the gene expression 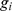 is the measure of relevance of that gene. Thus the maximum relevance condition is to maximize the average relevance of all genes in *s* is Equation 2:



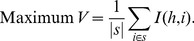
(2)Therefore, the redundancy of a gene has to be minimized and relevance of a gene has to be maximized. As two conditions are equally important, two simplest combined criteria are: 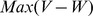, and 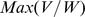. Here only the mRMR for discrete variable in form of mRMR mutual information quotient (mRMR MIQ) is described. The mRMR with MIQ scheme is formulated as per Equation 3.
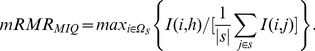
(3)


Next, in Graph-based feature selection method [36], a graph 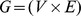 has been constructed with node-set *V*, edge-set 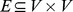 and edge weight matrix *W* whose elements are in the interval [0; 1]. Each vertex represents a feature and the edge between two features represents their pair wise relationship. The weight on the edge reflects the degree of relevance between two features. Therefore, the graph *G* with the corresponding edge-weight or weighted relevance matrix has been formed. The algorithm states: a) computing the relevance matrix 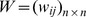 based on the mutual information between feature vectors, b) dominant-set clustering to cluster the feature vectors and c) selecting the optimal feature set from each dominant set using the multidimensional interaction information (MII) criterion. Therefore, in Cluster-based feature selection method [37], the feature set is partitioned into clusters of similar features where the number of clusters and the cardinality of the subset of selected features, is automatically estimated from the data. But this method relies on some user defined parameters.

### Multiobjective Optimization (MOO) and Problem Description

In this section first the basic concepts of multiobjective optimization are described. Subsequently, the formulation of gene selection problem as multiobjective optimization problem is described.

#### MOO concepts

In many real world problems, there exist different aspects of solutions which are partially or wholly in conflict. Therefore, treating those problems as single objective optimization produces an unreliable result. In multiobjective optimization problem the objectives may estimate those different aspects of solutions which are conflicting in nature. The multiobjective optimization can formally be stated as follows [18], [19]. Find the vector 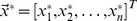
of decision variables which satisfies *m* inequality constraints:
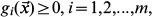
(4)and *p* equality constraints:
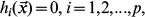
(5)and optimizes the vector function:



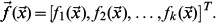
(6)The constraints in Equation 4 and 5 define the feasible region 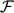 which contains all the allowable solutions. Any solution outside this region is inadmissible since it violates one or more constraints. The vector 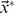 denotes an optimal solution in 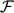.

The essence of multiobjective optimization technique can be determined through Pareto optimality [21]. Pareto optimal set comprises of all those solutions for which it is impossible to improve any objective without simultaneous worsening in some other objective. It can be said that a vector of decision variables 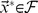 is Pareto optimal if there does not exist another 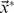 such that 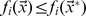 for all 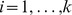 and 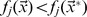 for at least one *j* when the problem is minimizing one. Here, 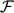 denotes the feasible region of the problem (i.e., where the constraints are satisfied). Pareto optimal set [22] generally contains more than one solution because there exist different ‘trade-off’ solutions to the problem with respect to different objectives. The set of solutions contained by Pareto optimal set are called non-dominated solutions. The plot of the objective functions whose non-dominated vectors are in the Pareto optimal set is called the Pareto front [22]. Specifically MOO is a process of generating the whole Pareto front or an approximation to it.

#### Problem description

**Figure 1:**
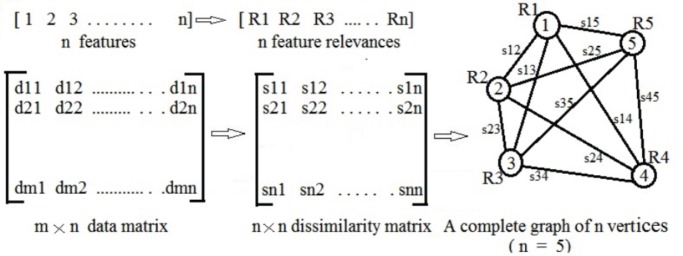
Construction of Feature-dissimilarity Graph. From the data matrix first Relevance Vector and Dissimilarity Matrix are Computed, then a weighted complete Feature-dissimilarity Graph is computed. Here an example of 5 feature-dissimilarity graph is depicted.



In this article the target is to find non-redundant but relevant features from a data matrix. In other words the resultant features are not only non-correlated but significant too. So the problem should be defined in such a manner that the correlated and irrelevant features are not selected. In our proposed scheme, the problem is equivalent to finding most dense subgraph from a weighted undirected graph. The arrangement of the data matrix can be viewed as a two-dimensional matrix where the rows indicate instances and columns indicate attributes or features. One additional column is there for presenting the corresponding class labels of the instances. A range of some similarity/dissimilarity measures includes correlation coefficient [38], Euclidean distance [39] and maximal information compression index [3] etc. Using one of these dissimilarity (negative similarity) measures the symmetric matrix is generated which is termed as a dissimilarity matrix. Let the data set has *n* features, 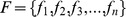. Calculating pairwise negative similarity between features of the feature set *F* manipulates 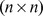 symmetric dissimilarity matrix *Sm*. Therefore from this dissimilarity matrix *Sm* a weighted complete graph *G* can be formed. Since a node represents a feature, so the vertex set of the graph *G* is 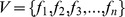, i.e., the graph contains total *n* nodes. The value at row *i* and column *j* in the dissimilarity matrix *Sm*, represents the weight of the edge between node 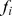 and 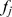. As each feature has some dissimilarity value with every other feature (present in dissimilarity symmetric matrix *Sm*), hence the graph *G* is a complete graph. Fig. 1 demonstrates the process of conversion from data matrix to feature-dissimilarity graph. First the dissimilarity matrix (for edge weight) is calculated for the data matrix using correlation coefficient between each pair of gene. The correlation coefficient 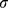 between two random variable *x* and *y* can be defined as [38]:

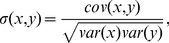
(7)where 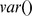 denotes the variance of a variable and 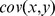 the covariance between the variables. If *x* and *y* are completely correlated, i.e., exact linear dependent exist, then 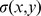 is 1 or −1 and if totally uncorrelated then 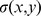 is 0. Hence 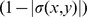 represents the dissimilarity between *x* and *y*. Subsequently, a graph *G* is formulated from the dissimilarity matrix. Let the samples are belong to either class1 (denoted by c1) or class2 (denoted by c2). Then the signal-to-noise ratio (SNR) value (node weight) corresponds to each feature (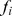) is calculated using mean and standard deviation (s.d.) of class1 samples (c1) and class2 samples (c2) and defined as [40]:



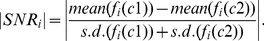
(8)The SNR describes the ratio of the relative mean to the sum of Standard Deviation of two classes of samples. Basically, it describes the difference between central tendency and variation or dispersion exists from the average value of the data points. A low SNR indicates that the feature does not have much different values in different classes. Whereas, high SNR indicates that the feature values are spread out over a large range of values and it is expected that the values are different in different classes. Very low SNR may be considered to be insignificant to the class labels and high SNR value means feature is highly differentially expressed. Therefore the SNR value is treated as feature relevance. For the graph *G* larger edge weight means that the features connected by that edge are more dissimilar and larger node weight means features are more relevant. Thus finding the most dense subgraph *g* from graph *G* is equivalent to finding the non-redundant and most relevant feature set, as the features (nodes) enclosed by the subgraph *g*, will have maximum average edge weight (dissimilarity) and maximum average node weight (SNR). Therefore the problem can be defined as: find the most densest subgraph (*g*) from a complete weighted graph *G*. Thus the features present in the reduced subgraph *g* are the required output of our proposed technique. Here we have developed a multiobjective bPSO to address this problem.

### Proposed Multiobjective Binary PSO-based Approach

Particle Swarm Optimization (PSO) [41], [42] is a well known swarm-based optimization techniques which optimizes a problem by iteratively trying to get better candidate solutions with respect to a given fitness measure. In PSO, a set of particles or candidate solutions traverse the search space with a velocity based on their own experience and the experience of their neighbors. During each traversal, the velocity and thereby the position of the particles are restructured. This process is repeated until some stopping criteria are met. Unlike other classical optimization techniques which tend to have premature convergence to local optimal solution, PSO is known for globalized searching.

In this article, the input data matrix is first transformed into a weighted undirected complete feature-graph, where the nodes (having relevance as node weight) symbolize the genes and the edges are weighted according to the dissimilarity of genes. In each iteration, a reduced subgraph is computed for which the average relevance and average dissimilarity among the genes contained by the reduced subgraph are maximized. Therefore, the densest subgraph having maximum average weight (node+edge) is identified by applying binary PSO [20]. The bPSO is applied to multiobjective optimization and with the help of non-dominated sorting [43] and Crowding Distance measure [18], small set of non-redundant informative genes is identified.

#### Particle encoding

Here the population is called swarm and it consists of *m* number of candidate solutions or particles. Each particle has *n* cells where *n* is the total number of genes comprises the data matrix i.e., each cell signify one gene from the data matrix. The cells can have values either 0 or 1. If the *i*-th cell of a particle has value 1 then *i*-th gene is selected from the dataset, otherwise it is ignored.

#### Initialization

Initially each cell of a particle is either 0 or 1 chosen randomly. After the initial particles are chosen, their corresponding fitness values are calculated. Then the velocity of each cell of the particle is initialized to zero. For each dataset, the algorithm is executed for 100 iterations. The input of the proposed system, i.e., the swarm size is set to 25 and the weighting factors c1 and c2 which are cognitive and social parameters respectively are set to 2.

#### Fitness computation

Here two objectives, average dissimilarity (negative correlation) and average signal-to-noise values are maximized. Each particle form a reduced subgraph for which average negative correlation 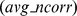 and average SNR value 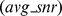 are computed. As the bPSO algorithm is designed as minimization problem, so fitness values are computed as 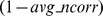 and 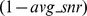. Then cells are iterated as usual PSO evaluation [44]. Now for calculating fitness values of a particle, those genes are selected for which representing cells have value 1. Therefore, these selected genes of the corresponding particle forms a subgraph 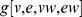 where *v* is the set of nodes, *e* is the set of edges, *vw* is a vector of node weights by computing SNR value for each node and *ew* is a edge weight matrix calculated by (1-correlation) between each pair of nodes. Thereafter, 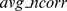 (Equation 9) and 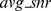 (Equation 10) are defined as
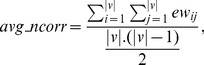
(9)




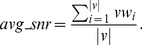
(10)


#### Updating position and velocity

As each cell represents one gene, so here the two terms cell and gene are used interchangeably. The position of a gene within a particle contains either 0 or 1, and velocity of each gene is initialized to zero. Using the information obtained from the previous step the position and velocity of each particle are updated. Each particle keeps track of the best position it has achieved so far in the history, and this best position is also called 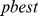 or local best. In multiobjective perspective, that position is chosen for 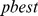 for which fitness of that particle dominates other fitnesses acquired by that particle in the history, if there is no such fitness then random choice is done between current and previous position of that particle. The best position among all the particles is called global best or 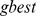 which is randomly chosen from the archive of non-dominated candidate solutions. Actually whenever a particle moves to a new position with a velocity, its position and velocity are altered according to the Equations 11 and 12 given below [20]:
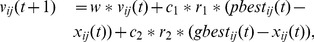
(11)




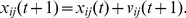
(12)


Here *t* is the time stamp and *i*-th particle and *j*-th position are considered. In Equation 11 new velocity (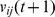) is acquired using velocity of previous time (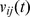), 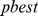 and 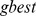. Then new position (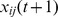) is obtained by adding new velocity with current position (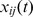) as shown in Equation 12. 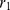 and 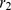 are two random value in the range of 0 to 1. 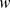 in Equation 13 is the inertia weight which is computed as:
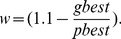
(13)


#### Updating archive

The repository where the non-dominated population in the history is reserved called archive. First the archive *A* is initialized with non-dominated population of 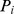. Next for updating the archive *A*, the next generation population 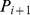 is merged with the archive 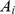 i.e., 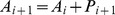 and then non-dominated solutions are yielded by applying non-dominated sorting and crowded distance sorting to the combined archive 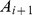. The non-dominated sorting and crowded distance sorting are evaluated for this combined population to obtain better diversity of the Pareto optimal front.

#### Proposed MObPSO algorithm

Here, the proposed multiobjective binary particle swarm optimization (MObPSO) is designed for maximizing the dissimilarity (negative correlation) and SNR, which are represented as edge weight and node weight, respectively. The adopted graph based MObPSO technique is illustrated in Table 1 Algorithm 1. The population is initialized by arbitrarily selected features from the data matrix and population fitness values are calculated using Equation 9 and Equation 10. The archive *A* is initialized by the population after non-dominated sorting of the primary population. Velocity and position are updated using Equations 11 and 12 respectively. Local best *P* is updated comparing the current fitness and previous fitness of a particle and global best *G* is updated according to random picking of particle from the archive. After updating the position and velocity, the archive is added with next generation solution and then non-dominated sorting [43] and crowding distance [18] sorting are used to revise the extended archive. These steps are repeated for particular number of iterations.

**Table 1:** Algorithm 1: Graph based MObPSO (Minimization Problem).

**Input**: data matrix 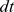 , 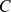 = number of genes, 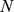 = number of particles, threshold 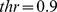 , Graph 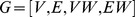 designed from dissimilarity matrix 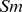 .
**Output**: archive 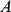
1: 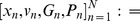 initialize( 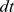 ) 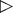 Random locations and velocities
2: 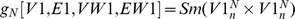 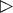 subgraphs 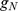 for 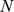 particles are formed from dissimilarity matrix 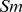
3: 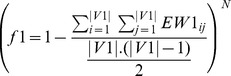 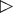 average dissimilarity value for the 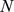 subgraphs
4: 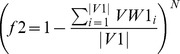 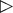 average snr value for the nodes contained by 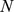 subgraphs
5: 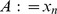 (if 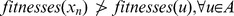 ) Initialize archive A by first non-dominated 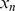
**6: for** 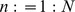 **do**
7: **for** 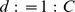 **do**
8: 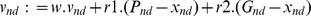
9: 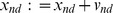
10: **if** 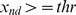 **then**
11: 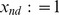 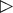 discretize the cell value
12: **else**
13: **if** 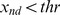 **then**
14: 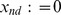
15: **end if**
16: **end if**
17: **end for**
18: **end for**
19: **for** 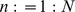 **do**
20: 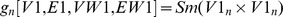 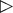 new subgraph produced by the evaluated particles
21: 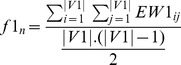 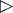 average dissimilarity value for the new subgraph
22: 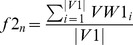 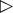 average snr value the nodes contained by the new subgraph
23: 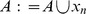 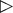 Add 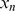 to 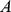
24: **for** 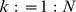 **do**
25: **if** 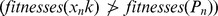 **then** 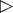 Update personal best
26: 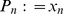
27: **if** Non-dominated fitnesses **then**
28: 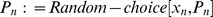
29: **end if**
30: **end if**
31: 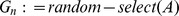
32: **end for**
33: **end for**
34: 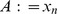 (if 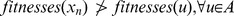 ) 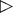 Non-dominated sorting is applied to the updated archive
35: CrowdingSort(A) 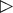 crowding distance sorting for archive
36: From step-6 to step-33 are repeated according to number of iteration

## Results and Discussion

In this section, we first describe the real-life datasets and their preprocessing procedure, thereafter portray the performance metrics followed by the results of different algorithms.

### Datasets and Preprocessing

In this article three real-life gene expression datasets are used which are publicly available from the following website: www.biolab.si/supp/bi-cancer/projections/info/.

#### Prostate

Gene expression measurements for samples of prostate tumors and adjacent prostate tissue not containing tumor were used to build this classification model. It contains 50 normal tissues and 52 prostate tumor samples. The expression matrix consists of 12533 number of genes and 102 number of samples.

#### DLBCL

Diffuse large B-cell lymphomas (DLBCL) and follicular lymphomas (FL) are two B-cell lineage malignancies that have very different clinical presentations, natural histories and response to therapy. Total 7070 genes are there in the dataset. The number of samples of type DLBCL is 58 and of type FL is 19.

#### GSE412 (Child-ALL)

The childhood ALL dataset (GSE412) includes gene expression information on 110 childhood acute lymphoblastic leukemia samples. The dataset has 50 examples of type before therapy and 60 examples of type after therapy. The number of genes is 8280.

The above described two-class datasets can be obtained as matrix format whose columns are genes and rows are samples and preprocessed by SNR (Equation 8) for each gene (column). The genes (column) of the data matrix are sorted according to the decreasing order of obtained 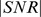. Lastly from the data matrix top 100 genes are taken. After that the data matrix is normalized to set each gene expression value in the range from 0 to 1.

### Score Analysis

Performance is evaluated using sensitivity, specificity, accuracy, fscore, AUC and average correlation. The entire dataset is divided into two different sets: training and test set. The proposed approach is applied on the training data. Therefore, a set of non-dominated candidate solutions are obtained. After that, for final marker genes assortment, we employ the 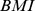-score [45] which considers the discriminative power of each gene by incorporating the true positive rate from logistic regression. In mathematical terms, let us assume a data set D consisting of two groups ‘control (ctr)’ and ‘experiment (exp)’. 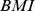 assigns a score for a feature 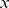 defined as follows:
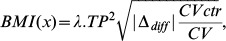
(14)where



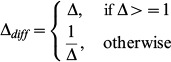
(15)Here, 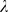 is a scaling factor and 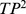 is the product of the true positive 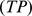 rates determined for each group using logistic regression. 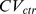 and 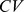 denote the coefficient of variance for the feature 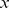 in the ‘control’ group and in both groups, respectively. Also, 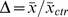, where 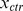, and 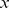 denote the mean value of 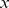 in ‘control’ and in both groups, respectively. The maximum 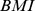-score generating candidate solution is considered as the most informative solution. The performance of the proposed algorithm is compared with that of its single objective versions and other two statistical tests like T-test and Wilcoxon Ranksum test. The datasets are arbitrarily divided into two sets: training set and test set. This process is repeated 10 times and we got 10 train sets and their corresponding 10 test sets. Each of the algorithms is executed for each train file and evaluated with the corresponding test file. Thus for each algorithm, we got 10 sensitivity, 10 specificity, 10 accuracy and 10 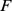-score values. Now the average of these 10 values for each performance metric with standard deviation are computed and tabularized.

With respect to Prostate data, it is evident from Table 2 that for each score metric the proposed method outperforms (0.8962, 0.9, 0.898, 0.9002, 0.964) the singleobjective versions, T-test, Ranksum test, SFS and SBE. Regarding sensitivity, our method is better than Graph-based and Cluster-based but differs slightly with CFS and mRMR (miq). Again with respect to specificity, the performance is average. In case of accuracy and fscore, proposed method is better than mRMR (miq) and Cluster-based method but not as good as CFS and Graph-based method. The AUC produced by the proposed method is 0.964 which is better than all the other methods. Except T-test, our method produces 0.4714 as average correlation which is less compared to that for the other methods. This indicates that non-correlated genes are identified. As population-based optimization techniques take more time to execute, therefore time complexity of our method is 81.176 Sec. which is not so high than other comparative methods.

**Table 2:** Performance Analysis for Three Real-life Data Set.

Dataset	Algorithm	Sensitivity	Specificity	Accuracy	Fscore	AUC	Average	Time
		(sd)	(sd)	(sd)	(sd)		correlation	(Sec.)
Prostate	Proposed	0.8962	0.9	0.898	0.9002	0.964	0.4714	81.176
Cancer		(0.0575)	(0.0909)	(0.047)	(0.0434)			
dataset	Singleobjective	0.8221	0.855	0.8382	0.8382	0.913	0.6343	45.39
	(correlation)	(0.0768)	(0.1024)	(0.06)	(0.0582)			
	Singleobjective	0.8701	0.865	0.8676	0.8704	0.9219	0.6646	53.72
	(SNR)	(0.0352)	(0.0563)	(0.0174)	(0.0142)			
	T-test	0.7778	0.8244	0.8497	0.8336	0.7817	0.4434	27.199
		(0.1462)	(0.0615)	(0.074)	(0.1052)			
	Ranksum test	0.8547	0.8375	0.8768	0.8522	0.8311	0.5177	23.267
		(0.0976)	(0.0825)	(0.0399)	(0.0317)			
	SFS	0.7393	0.7864	0.7950	0.7126	0.7308	0.5514	21.382
		(0.1272)	(0.2313)	(0.1047)	(0.0847)			
	SBE	0.78	0.7116	0.763	0.7233	0.7701	0.612	46.113
		(0.1882)	(0.2839)	(0.179)	(0.0787)			
	CFS	0.9131	0.9001	0.9112	0.9211	0.9215	0.4993	73.9
		(0.061)	(0.0672)	(0.0561)	(0.0373)			
	mRMR(miq)	0.9176	0.8686	0.8936	0.8970	0.9484	0.579	64.83
		(0.0783)	(0.0552)	(0.0564)	(0.0574)			
	Graph-based	0.8646	0.96	0.9216	0.92	0.9362	0.4773	39.172
		(0.061)	(0.197)	(0.048)	(0.528)			
	Cluster-based	0.8077	0.9211	0.8431	0.84	0.92	0.4815	23.18
		(0.0593)	(0.093)	(0.0514)	(0.0512)			
DLBCL	Proposed	0.9111	0.9207	0.9184	0.8428	0.9644	0.6128	113.81
dataset		(0.1021)	(0.0564)	(0.0315)	(0.0513)			
	Singleobjective	0.6389	0.8966	0.8355	0.639	0.8167	–	63.11
	(correlation)	(0.24)	(0.0922)	(0.07)	(0.148)			
	Singleobjective	0.8333	0.8707	0.8618	0.7434	0.9214	0.6369	79.443
	(SNR)	(0.0197)	(0.1219)	(0.0767)	(0.1082)			
	T-test	0.7284	0.9119	0.8486	0.7052	0.6672	0.6667	43.78
		(0.3148)	(0.0881)	(0.1036)	(0.268)			
	Ranksum test	0.7654	0.8945	0.8621	0.7327	0.4252	0.778	51.9
		(0.2747)	(0.0622)	(0.0668)	(0.1991)			
	SFS	0.5714	0.7783	0.7293	0.5997	0.4760	0.6767	87.76
		(0.2437)	(0.1318)	(0.0921)	(0.1558)			
	SBE	0.6814	0.7000	0.6744	0.6119	0.60	0.7011	96.221
		(0.2773)	(0.218)	(0.201)	(0.2056)			
	CFS	0.5556	0.9355	0.8684	0.6667	0.9308	0.5101	51.482
		(0.0233)	(0.0354)	(0.0424)	(0.021)			
	mRMR(miq)	0.8889	0.9163	0.9098	0.8244	0.9568	0.639	80.11
		(0.0641)	(0.0337)	(0.0298)	(0.0544)			
	Graph-based	0.7889	1	0.9337	0.8402	0.8462	0.4413	43.91
		(0.0367)	(0.0493)	(0.0291)	(0.0552)			
	Cluster-based	0.8779	0.8966	0.8947	0.8	0.9637	0.5815	56.225
		(0.0339)	(0.0601)	(0.0441)	(0.052)			
Child-ALL	Proposed	0.752	0.8233	0.7909	0.7671	0.8639	0.7324	81.3
dataset		(0.0648)	(0.1055)	(0.053)	(0.0501)			
	Singleobjective	0.71	0.8042	0.7614	0.7295	0.743	–	66.96
	(correlation)	(0.0763)	(0.0844)	(0.0382)	(0.0414)			
	Singleobjective	0.64	0.8442	0.7568	0.7079	0.8073	0.7854	71.014
	(SNR)	(0.0676)	(0.1296)	(0.0701)	(0.0692)			
	T-test	0.4960	0.68	0.5964	0.5184	0.8253	0.9014	21.681
		(0.1943)	(0.3719)	(0.1596)	(0.1728)			
	Ranksum test	0.4640	0.87	0.6855	0.5506	0.8114	0.9223	23.575
		(0.2296)	(0.2755)	(0.1108)	(0.1901)			
	SFS	0.46	0.8556	0.6758	0.5402	0.84	0.7416	63.014
		(0.1908)	(0.1089)	(0.0656)	(0.1766)			
	SBE	0.6878	0.6173	0.5889	0.6202	0.84	0.7655	71.224
		(0.2108)	(0.29)	(0.0197)	(0.2689)			
	CFS	0.6400	0.9133	0.789	0.7442	0.8427	0.6313	76.44
		(0.191)	(0.1189)	(0.06114)	(0.0677)			
	mRMR(miq)	0.7486	0.8762	0.7782	0.7896	0.8802	0.741	69.886
		(0.0380)	(0.0600)	(0.0315)	(0.0313)			
	Graph-based	0.44	1	0.7455	0.6111	0.9267	0.7813	52.13
		(0.055)	(0.21)	(0.0671)	(0.0519)			
	Cluster-based	0.749	0.8164	0.7818	0.7917	0.8133	0.7399	59.45
		(0.061)	(0.093)	(0.0409)	(0.0572)			

For DLBCL data, Table 2 shows that with respect to average sensitivity, fscore and AUC our proposed technique (0.9111, 0.8428 and 0.9644) uniformly scores better than all the other methods. With respect to specificity, proposed method has scored better than all but CFS and Graph-based method. The accuracy produced by our method is also better than others except Graph-based method. Although CFS and Graph-based method result less correlated genes but their sensitivity is very bad. Time complexity for proposed method is higher than others but however, the difference is not very high.

Moreover, for Child-ALL data, it is clear from Table 2 that the proposed scheme has established its superiority in case of sensitivity, accuracy. But with respect to average specificity the score is 0.8233 which is not better than singleobjective (SNR), Ranksum test, SFS, CFS, mRMR (miq) and Graph-based method. But with respect to fscore and AUC, most of the time, proposed method produce better score than others. Again average correlation of the proposed method is 0.7324 which is also the lower than others except CFS. Hence the proposed technique uniformly yields better values which prove the superiority of our proposed technique.

### Cross-Validation Performance

The performance analysis is extended using 10-fold cross validation. All the algorithms are executed on the total sample versus gene dataset and the output genes are validated using 10-fold cross-validation using Support Vector Machine (SVM). The cross-validation scores of different algorithms are reported in Table 3. It is clear from the table that for the prostate dataset, with respect to sensitivity, specificity, accuracy and fscore proposed method outperforms than other methods except CFS. With respect to AUC, our method is better than CFS, mRMR(miq), Graph-based and Cluster-based. The average correlation for our method is very much lower than other methods i.e. proposed method results more non-redundant features than other comparative methods. But it is obvious from the table that it took more time to execute than others. In case DLBCL dataset, with respect to sensitivity, accuracy, fscore and AUC, the proposed method performs best among all the methods. With respect to specificity, the proposed method performs slightly less than singleobjective (SNR), T-test, Ranksum test, CFS and Graph-based method. The average correlation produces by the proposed technique is less than other methods except mRMR (miq). It can also be noticed from the table that the execution time for the proposed method is 3.6832 Seconds but the difference with other method is less. For the Child-ALL dataset, with respect to accuracy, fscore and AUC the proposed method performs better than other comparative methods. With respect to sensitivity, the score is average and less than other methods. The specificity scored by the proposed technique is 0.719 which highly better than other methods except Graph-based method. The proposed method produced 0.6764 as average correlation which is less than other methods except CFS.

**Table 3:** 10-fold Cross-validation Result Analysis for Three Real-life Data Set.

Dataset	Algorithm	Sensitivity	Specificity	Accuracy	Fscore	AUC	Average correlation	Time (In Sec.)
								
Prostate Cancer	Proposed	0.9423	0.9515	0.9412	0.9423	0.9624	0.4136	4.1278
dataset	Singleobjective	0.9038	0.82	0.8627	0.8704	0.9505	0.4770	1.6487
	Singleobjective	0.8846	0.94	0.9118	0.9109	0.9415	0.4743	1.643
	T-test	0.9142	0.9419	0.9336	0.9314	0.9554	0.5367	1.184
	Ranksum test	0.8846	0.9344	0.9216	0.92	0.9485	0.5203	1.094
	SFS	0.819	0.8741	0.91	0.8901	0.8531	0.476	1.031
	SBE	0.7951	0.8146	0.8863	0.8359	0.8322	0.4993	1.8351
	CFS	0.9231	0.96	0.951	0.9401	0.9835	0.4211	1.8989
	mRMR(miq)	0.9387	0.93	0.9351	0.9412	0.9665	0.3929	1.362
	Graph-based	0.9231	0.94	0.9314	0.932	0.967	0.4743	2.189
	Cluster-based	0.9038	0.94	0.9216	0.9216	0.96	0.5245	3.0718
DLBCL	Proposed	1	0.9483	0.961	0.9268	0.9955	0.4658	3.6832
	Singleobjective	0.8421	0.9377	0.9221	0.8421	0.9737	0.4926	2.5195
	Singleobjective	0.6263	0.9655	0.9551	0.6452	0.961	0.5692	2.4434
	T-test	0.8153	0.965	0.9366	0.8649	0.9837	0.5412	1.233
	Ranksum test	0.8944	0.951	0.949	0.9231	0.9946	0.5152	1.391
	SFS	0.7222	0.898	0.8318	0.834	0.868	0.5016	1.05
	SBE	0.6319	0.9133	0.8001	0.7822	0.823	0.4772	1.7312
	CFS	0.9474	0.9518	0.949	0.9191	0.9894	0.4408	2.47
	mRMR(miq)	0.9474	0.9432	0.9487	0.9231	0.9809	0.4545	1.898
	Graph-based	0.8947	0.9601	0.9481	0.8947	0.9827	0.5538	2.43
	Cluster-based	0.7105	0.9383	0.7662	0.8077	0.8818	0.5517	3.3227
Child-ALL	Proposed	0.8813	0.719	0.8733	0.7352	0.8801	0.6764	3.6605
	Singleobjective	1	0.0167	0.4638	0.6289	0.796	0.7576	3.22
	Singleobjective	0.989	0.1701	0.4545	0.625	0.8133	0.6926	2.92
	T-test	0.82	0.4333	0.6091	0.656	0.8427	0.73	2.73
	Ranksum test	0.80	0.45	0.6091	0.6504	0.8060	0.7136	2.899
	SFS	0.9667	0.2961	0.4358	0.7111	0.828	0.6936	2.39
	SBE	1	0.017	0.4913	0.6535	0.783	0.725	2.873
	CFS	0.9411	0.0677	0.4833	0.6144	0.8403	0.5989	3.3486
	mRMR(miq)	0.7	0.4167	0.5455	0.5833	0.692	0.6774	2.968
	Graph-based	0.6909	0.7333	0.7455	0.7143	0.7297	0.6786	3.43
	Cluster-based	0.94	0.1851	0.4818	0.6395	0.7927	0.6817	3.6357

### Gene Marker Analysis

**Figure 2:**
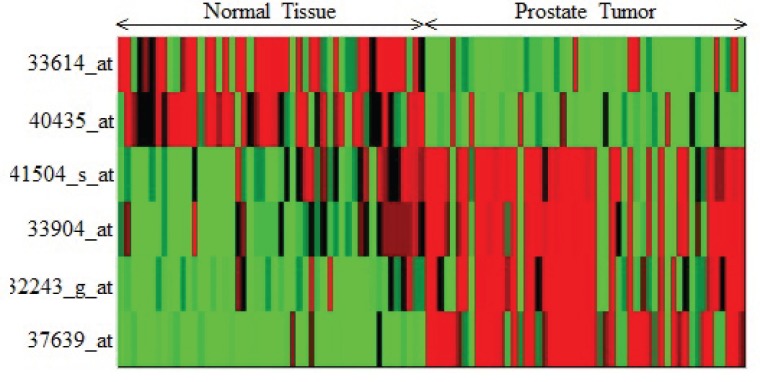
The Heatmap of the gene markers for Prostate Cancer data. The Heatmap describe the expression levels of the four up-regulated and two down-regulated gene markers for normal and cancerous type in Prostate Cancer data.

**Figure 3:**
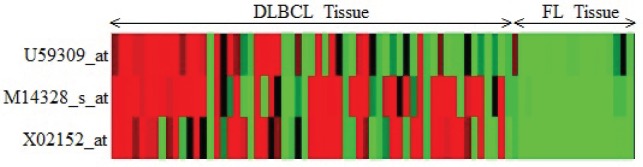
The Heatmap of the gene markers for DLBCL data. The Heatmap describe the expression levels of the three down-regulated gene markers for DLBCL and FL type in DLBCL data.

**Figure 4:**
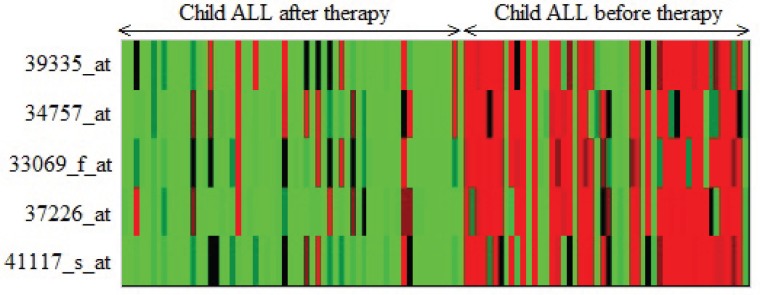
The Heatmap of the gene markers for Child-ALL data. The Heatmap describe the expression levels of the five down-regulated gene markers for after and before therapy in Child-ALL data.

After executing the proposed technique 10 times we got 10 feature sets. Thereafter we took those genes as maker which appears at least 5 times in the 10 feature sets. Table 4 describes the gene markers ID, Symbol and Description for the three datasets. Among the gene markers, many of those have already been validated to be associated with the respective cancer classes in different existing literature. Such as for prostate cancer data the gene 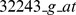 (CRYAB) and 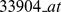 (CLDN3) have been reported in [46] and 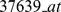 (HPN) and 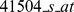 (MAF) have been reported in [47]. Also the genes 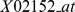 (LDHA) and 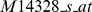 (ENO1) of DLBCL have been reported in [48]. Again in [49], the genes 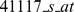 (SLC9A3R2), 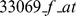 (UGT2B15)of Child-ALL data are reported. In Fig. 2, Fig. 3 and Fig. 4, the heatmaps of the feature sets identified by our proposed technique for prostate dataset, DLBCL dataset and child-all dataset are shown respectively. The heatmaps show gene versus sample matrix. The cells of the heatmap represent the expression levels of the genes in terms of colors. The red shades represent high expression levels whereas the green shades represent low expression levels and the colors towards black represent the medium expression values. It is evident from the figures (2, 3 and 4) that the gene markers for each tumor subtype has either high expression values (Up-regulated) or low expression values (Down-regulated) over all the samples of the respective tumor class. From Fig. 2, it is clear that the genes 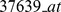 (HPN), 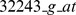 (CRYAB), 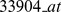 (CLDN3) and 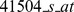 (MAF) are up-regulated (high expression value in normal tissue and low expression in tumor tissue) and genes 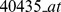 (SLC25A6) and 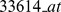 (RPL18A) are down-regulated (vice-versa). Then it can be seen from Fig. 3 that the genes 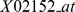 (LDHA), 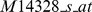 (ENO1) and 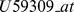 (FH) are all down-regulated with respect to DLBCL to FL. Subsequently, for child-ALL data all genes are down-regulated because Fig. 4 depicts that high expression value in before-therapy class and low expression value in after-therapy class.

**Table 4:** Gene Markers Identified by the Proposed Method for Various Dataset.

Data set	Gene ID	Symbol	Description	Up or Down
				
**Prostate**	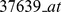	HPN	Hepsin	up
**Cancer**	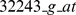	CRYAB	crystallin, alpha B	up
	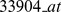	CLDN3	claudin 3	up
	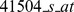	MAF	v-maf musculoaponeurotic fibrosarcoma oncogene homolog	up
	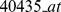	SLC25A6	solute carrier family 25, member 6	down
	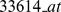	RPL18A,	ribosomal protein L18a, L18a pseudogene 3	down
		RPL18AP3		
**DLBCL**	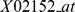	LDHA	lactate dehydrogenase	down
	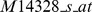	ENO1	enolase 1 (alpha)	down
	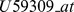	FH	fumarate hydratase, mitochondrial precursor	down
**Child**	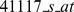	SLC9A3R2	solute carrier family 9, isoform 3 regulator 2	down
**ALL**	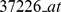	BNIP1	BCL2/adenovirus E1B 19 KDa interacting protein 1	down
	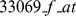	UGT2B15	UDP glucuronosy1transferase 2 family, polypeptide B15	down
	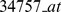	PARP2	poly (ADP-ribose) polymerase 2	down
	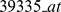	EIF5AL1,	eukaryotic translation initiation factor 5A-like1 and 5A	down
		EIF5A		

## Conclusion

In this proposed study, the problem of supervised feature selection is posed as relevant and non-redundant gene markers identification from microarray gene expression data. The microarray data matrix has been converted into feature-dissimilarity graph where nodes stand for features. The nodes and edges are weighted according to feature relevance and dissimilarity value between features, respectively. Then the densest subgraph having maximum average node and edge weight has been identified that means features with high relevance and less redundant are selected as output. For identifying subgraph having non-redundant and relevant feature nodes, a graph based multiobjective bPSO has been proposed. Here, bPSO has been modeled using multiobjective framework which is based on non-dominated sorting and crowding distance sorting. Three real life datasets have been used for performance analysis. The comparative study between the proposed technique and its single objective versions, T-test and Ranksum test has been performed. Moreover, gene marker analysis with respect to each dataset is also illustrated. As a future scope, we plan to incorporate a supervised wrapper based approach to calculate objective functions using fuzzy association rules.
